# Intranasal Delivery of Gallium–Quercetin Nanoparticles for Multi‐Target Ferroptosis Inhibition in Parkinson's Disease

**DOI:** 10.1002/advs.77025

**Published:** 2026-08-07

**Authors:** Keyang Xu, Dandan Kou, Xiao Xiao, Bingbing Bai, Caide Liu, Mengyao Wu, Yadan Shi, Mengdan Xu, Xiaoyi Cao, Haodi Qi, Shuhua Wu, Jianfeng Zeng, Mingyuan Gao

**Affiliations:** ^1^ Department of Radiology The First Affiliated Hospital of Soochow University School of Radiation Medicine and Protection Soochow University Suzhou China; ^2^ General Dentistry Department The Stomatology Hospital Affiliated of Suzhou Vocational Health College Suzhou China; ^3^ Medical School of Nanjing University Nanjing China; ^4^ The Second Affiliated Hospital of Soochow University Suzhou China; ^5^ Biomedical Basic Research Center (BBRC) of Jiangsu Province Suzhou China; ^6^ School of Life Sciences Soochow University Suzhou China

**Keywords:** ferroptosis inhibition, gallium quercetin nanoparticles, intranasal drug delivery, iron metabolism regulation, Parkinson's disease therapy

## Abstract

Ferroptosis contributes to Parkinson's disease (PD) through interconnected processes including iron dysregulation, oxidative stress, and mitochondrial dysfunction, yet current therapies targeting single pathways remain insufficient. Herein, we developed gallium–quercetin nanoparticles (GQNPs) as an intranasally deliverable nanoplatform for multi‐target ferroptosis inhibition. In vitro, GQNPs suppressed ferroptosis by coordinating iron regulation and antioxidation. Ga^3^^+^ interfered with transferrin‐mediated iron uptake to restrict iron influx, while quercetin reduced oxidative stress and supported iron homeostasis, thereby decreasing ROS accumulation and improving mitochondrial function. In vivo, intranasal delivery of GQNPs effectively bypassed the blood‐brain barrier to recover motor coordination and cognitive function in PD mice. By integrating iron regulation, antioxidant activity, and mitochondrial protection within a single nanoplatform, this work highlights gallium‐based coordination nanoparticles as a promising therapeutic strategy for ferroptosis‐associated neurodegenerative diseases.

## Graphical abstract

Leveraging the chemical similarity between Ga^3+^ and Fe^3+^ as well as the antioxidant properties of quercetin, gallium–quercetin nanoparticles (GQNPs) were prepared to integrate iron homeostasis regulation, oxidative stress suppression, and mitochondrial protection for multi‐target ferroptosis inhibition in Parkinson's Disease.

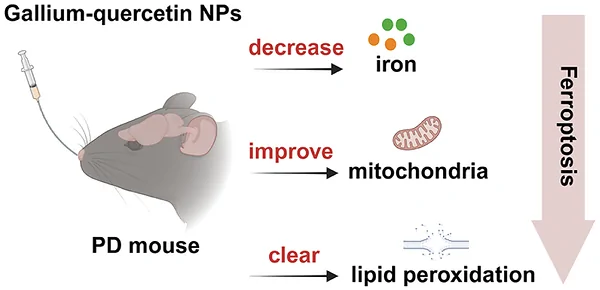

**Abbreviations**
ALB: albumin
ALT: aminotransferase
BBB: blood‐brain barrier
CCK8: Cell Counting Kit‐8
CLSM: confocal laser scanning microscopy
CNS: central nervous system
CREA: creatinine
DFP: deferiprone
DMT1: divalent metal transporter 1
ELISA: enzyme‐linked immunosorbent assay
FBS: fetal bovine serum
Fe^3+^
: ferric ion
FPN: ferroportin
FTH1: ferritin heavy chain 1
Ga^3+^
: Gallium ion
GPX4: glutathione peroxidase 4
GQNPs: gallium–quercetin nanoparticles
GSH: glutathione
H&E: Hematoxylin‐eosin
HGB: hemoglobin
ICP: inductively coupled plasma
L‐DOPA: levodopa
MDA: Malondialdehyde
MPP^+^
: 1‐methyl‐4‐phenylpyridinium
NRF2: nuclear factor erythroid 2‐related factor 2
PD: Parkinson's disease
PLT: hemoglobin
PVP: polyvinylpyrrolidone
RBC: red blood cell
SNpc: substantia nigra pars compacta
TEM: transmission electron microscopy
TFR1: transferrin receptor 1
TH: tyrosine hydroxylase
TP: total protein
WBC: white blood cell

## 1 Introduction

Parkinson's disease (PD) is a progressive neurodegenerative disorder characterized by the selective degeneration of dopaminergic neurons in the substantia nigra pars compacta (SNpc) [1]. The resulting dopamine (DA) deficiency in the striatum leads to typical motor symptoms, including bradykinesia, rigidity, resting tremor, and postural instability, along with various non‐motor symptoms that substantially impair patients’ quality of life [2]. Given the progressive nature and debilitating effects of PD, the development of effective therapy remains an urgent clinical need.

The current clinical treatment for PD relies on dopaminergic replacement with levodopa (L‐DOPA) [3]. L‐DOPA can cross the blood‐brain barrier (BBB) and be converted into DA by aromatic L‐amino acid decarboxylase, thereby alleviating motor symptoms [4]. However, its long‐term efficacy is limited by the continuous loss of dopaminergic neurons, which makes dose adjustment increasingly necessary and may contribute to L‐DOPA‐induced dyskinesia [5]. Adjunct therapies have also been developed to improve symptomatic control, including dopamine agonists (e.g., pramipexole and ropinirole), which activate dopamine receptors, and monoamine oxidase‐B inhibitors (e.g., selegiline and rasagiline), which reduce DA degradation [6]. With respect to advanced PD, deep brain stimulation has been developed to improve motor symptoms by modulating dysfunctional neural circuits [7, 8]. However, these therapeutic approaches primarily target dopaminergic signaling and do not effectively prevent the progressive degeneration of dopaminergic neurons. Therefore, therapeutic strategies that directly interfere with the underlying neurodegenerative process are highly desirable.

Although the precise mechanisms of PD remain incompletely understood, multiple pathological processes have been implicated in disease progression, including α‐synuclein aggregation [9], mitochondrial dysfunction [10], neuroinflammation [11, 12], oxidative stress [13], and dysregulated iron homeostasis [14, 15]. Among these factors, ferroptosis, an iron‐dependent form of regulated cell death, has recently been identified as a key contributor to dopaminergic neurodegeneration in PD [16]. Elevated iron levels, oxidative stress, and lipid peroxidation, which are the central features of ferroptosis, are all closely associated with PD pathology [17]. Dopaminergic neurons are particularly vulnerable to ferroptotic damage because of their high metabolic demand, extensive axonal projections, and dependence on mitochondrial energy production. Mitochondrial ATP generation requires iron‐containing proteins, whereas excessive iron accumulation can disturb mitochondrial function, increase membrane potential, and exacerbate ROS generation [18, 19]. In addition to mitochondrial impairment, iron overload can induce protein oxidation and endoplasmic reticulum stress, further increasing oxidative stress [20]. Fe^2+^ also participates in the Fenton reaction, generating hydroxyl radicals that directly oxidize polyunsaturated fatty acids and initiate lipid peroxidation [21]. Under physiological conditions, the GPX4‐GSH axis reduces lipid peroxides to lipid alcohols and thereby suppresses ferroptosis [22]. However, reduced antioxidant enzyme levels in the substantia nigra, together with the high lipid content and oxygen consumption, render dopaminergic neurons highly susceptible to oxidative injury [23]. Moreover, dysregulated interactions between iron ions and iron‐responsive proteins can alter the expression of iron‐regulatory proteins and exacerbate α‐synuclein aggregation in PD [24]. Recent studies suggest that ferroptosis precedes apoptosis, as its inhibition can prevent subsequent apoptotic events [25]. These findings indicate that restoring iron homeostasis and suppressing ferroptosis may represent a promising therapeutic approach.

Despite the therapeutic potential, current ferroptosis‐targeted strategies remain limited. Iron chelators, such as deferiprone (DFP), can reduce iron overload but may cause excessive iron depletion and systemic toxicity [26]. Ferroptosis inhibitors, including ferrostatin‐1 and liproxstatin‐1, have shown neuroprotective effects, but their limited specificity and poor brain availability restrict their clinical application [19]. Recently, nanomedicine‐based strategies for ferroptosis inhibition have attracted increasing attention because of their ability to modulate iron metabolism or oxidative stress while improving brain delivery [14, 27, 28]. However, most reported strategies mainly focus on a single pathway and have difficulty simultaneously regulating iron influx, iron‐homeostasis‐related proteins, oxidative stress, and mitochondrial dysfunction. Considering the complex pathophysiology of PD, a multi‐target strategy is preferable for effectively suppressing ferroptosis‐associated neurodegeneration.

Gallium ion (Ga^3+^) and ferric ion (Fe^3+^) share similar ionic radii, ionization potentials, and electron affinities, allowing Ga^3+^ to function as a redox‐inert iron substitute [29]. Previous studies have shown that Ga^3+^ can decrease iron content by interfering with iron uptake, thereby exhibiting antibacterial and anti‐cancer properties [30, 31]. However, the role of Ga^3+^ in ferroptosis appears to be context‐dependent. For example, a salen‐Ga complex was reported to disrupt mitochondrial function and impair the glutathione antioxidant system, eventually triggering ferroptosis in cancer cells [32]. In contrast, a Ga^3+^/gallic acid complex was shown to inhibit ferroptosis in kidney injury [33]. These findings suggest that gallium‐based nanoparticles may provide a tunable platform for ferroptosis regulation. Nevertheless, the standalone use of Ga^3+^ may induce unintended detrimental effects [34]. Therefore, polyphenols such as quercetin were selected to coordinate with Ga^3+^, regulate its release, and provide complementary antioxidant activity.

Quercetin is a naturally occurring flavonoid with well‐established antioxidant and anti‐inflammatory properties. Accumulating evidence suggests that its role in ferroptosis is context‐dependent. In some cancer models, quercetin can promote ferroptosis through autophagy‐related pathways, thereby suppressing tumor growth [35]. By contrast, in non‐cancerous pathological conditions, such as kidney injury, liver injury, and neurodegenerative disorders including PD, quercetin has been reported to exert anti‐ferroptotic effects by activating the NRF2/GPX4 signaling axis, enhancing cellular antioxidant capacity and mitigating tissue damage [36]. Ferroptosis regulation is also closely associated with mitochondrial quality control. For example, carvedilol has been shown to alleviate ferroptosis in a PD model by promoting mitophagy through the PINK1/PARKIN pathway [37]. Similarly, quercetin has been reported to enhance mitochondrial autophagy via PINK1 signaling in kidney fibrosis [38]. These findings suggest that quercetin may suppress ferroptosis not only through redox regulation but also by mitochondrial protection. Moreover, quercetin has been reported to chelate iron in PD models, further supporting its multifunctional role in ferroptosis modulation. Taken together, these properties make quercetin a rational ligand to complement Ga^3+^. The combination of Ga^3+^ and quercetin is therefore expected to provide cooperative therapeutic effects by integrating iron regulation, antioxidant defense, and mitochondrial protection, while introducing a gallium‐based strategy for ferroptosis inhibition in PD.

Herein, we report gallium–quercetin nanoparticles (GQNPs) as an intranasally deliverable nanoplatform for multi‐target ferroptosis inhibition in PD. As shown in Scheme 1, GQNPs were designed to modulate iron metabolism, alleviate oxidative stress, and preserve mitochondrial function by integrating the complementary activities of Ga^3+^ and quercetin. Owing to its chemical similarity to Fe^3+^, Ga^3+^ was expected to interfere with transferrin‐mediated iron uptake, thereby reducing iron influx and alleviating iron‐associated toxicity. Meanwhile, quercetin served not only as a coordination ligand but also as an active therapeutic component with antioxidative and anti‐inflammatory properties. Beyond evaluating the overall anti‐ferroptotic efficacy of GQNPs, we further investigated their effects on key molecular regulators of ferroptosis, including iron‐homeostasis‐related proteins, oxidative stress markers, and mitochondrial function. The therapeutic efficacy of GQNPs was then evaluated following intranasal administration (Figure S1). The current study demonstrates the neuroprotective effects of GQNPs both in vitro and in vivo, supporting the feasibility of multi‐target intervention as a nanomedicine‐based therapeutic strategy for PD.

**SCHEME 1:**
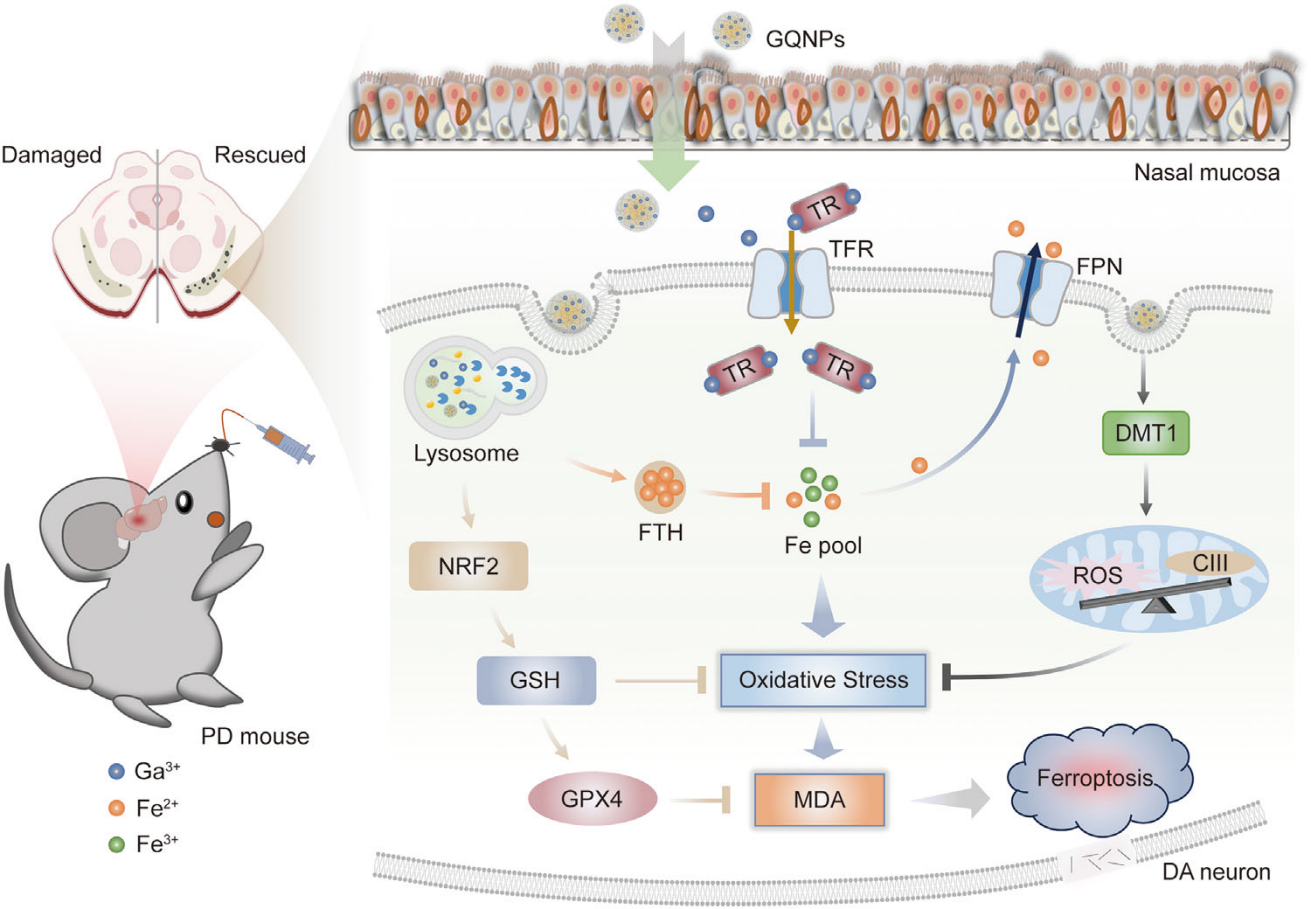
Schematic illustration of the therapeutic mechanism of GQNPs in PD through multi‐target ferroptosis inhibition. Following intranasal administration, GQNPs are delivered to the brain and suppress ferroptosis through coordinated regulation of iron metabolism, oxidative stress, and mitochondrial function. Ga^3+^ interferes with transferrin‐mediated iron uptake and modulates iron‐homeostasis‐related proteins, including TFR1 and FTH1, thereby limiting iron influx and reducing the intracellular labile iron pool. Meanwhile, the quercetin component exerts antioxidant effects by scavenging ROS, activating NRF2 signaling, enhancing GPX4 expression, and inhibiting lipid peroxidation. In addition, GQNPs alleviate mitochondrial dysfunction by reducing mitochondrial ROS and preserving mitochondrial respiratory chain complex III activity. Together, these coordinated effects enable GQNPs to inhibit ferroptosis and protect dopaminergic neurons in PD.

## 2 Results

### 2.1 Synthesis and Characterization of GQNPs

Quercetin is a polyphenolic compound with a strong ability to chelate metal ions to form stable coordination structures. Previous studies have demonstrated that polyvinylpyrrolidone (PVP) can regulate the self‐assembly of polyphenol–metal complexes, yielding nanoparticles with controllable sizes and improved water dispersibility. Based on these studies [39, 40], GQNPs were prepared through a straightforward method. As illustrated in Figure 1a, PVP and quercetin were first dissolved in methanol, followed by the dropwise addition of Ga(NO_3_)_3_ solution in methanol. This process resulted in a color change from light yellow to orange, suggesting the occurrence of coordination interactions between quercetin and Ga^3+^ (Figure 1b). UV–vis absorbance spectroscopy revealed that mixing quercetin with Ga^3+^ generated a new absorption band at approximately 475 nm, accompanied by the disappearance of the original quercetin absorption at around 375 nm (Figure 1c), indicating the formation of a quercetin–gallium coordination complex. In addition, the appearance of fluorescence peaking at approximately 600 nm further supported the successful formation of the coordination complex (Figure 1d).

**FIGURE 1:**
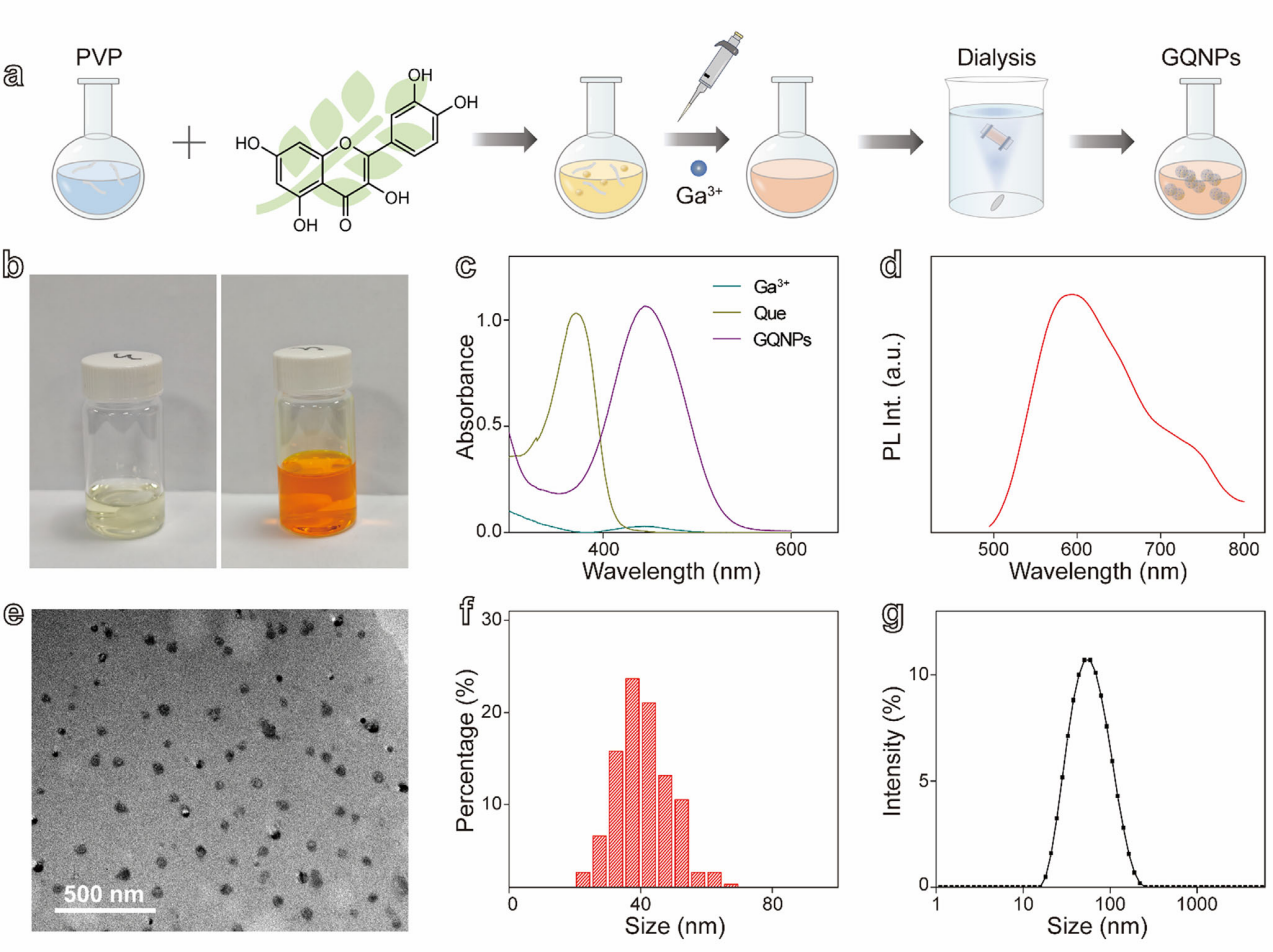
Synthesis and characterization of GQNPs. (a) Schematic illustration of the synthesis of GQNPs, (b) representative images showing the color change of the quercetin solution before and after the addition of Ga^3+^ in the presence of PVP, (c) UV–vis absorption spectra of Ga^3+^, quercetin, and GQNPs, (d) fluorescence emission spectrum of GQNPs, (e) TEM image of GQNPs, (f) size distribution of GQNPs based on the TEM image shown in panel e, (g) hydrodynamic size distribution of GQNPs in water.

PVP was introduced into the reaction system to promote the formation of nanoparticles from the resulting complexes. Transmission electron microscopy (TEM) results showed that the nanoparticles had an average diameter of 40.5 ± 10.1 nm (Figure 1e,f), while the hydrodynamic size of GQNPs in pure water was around 51.1 nm (Figure 1g). Furthermore, GQNPs maintained good colloidal stability in phosphate‐buffered saline (PBS) and 10% fetal bovine serum (FBS) (Figure S2a). The gradual decrease in particle size observed in PBS and FBS may be attributed to a gradual degradation of nanoparticles in biological media. The zeta potential of GQNPs was −4.27 mV, indicating a slightly negative surface charge, which may facilitate nose‐to‐brain delivery by reducing excessive interactions with negatively charged nasal mucus and thereby enhancing diffusion (Figure S2b).

The antioxidant capacity of GQNPs was assessed using a total antioxidant capacity assay kit based on the ABTS method. Gallium nitrate alone showed negligible ROS‐scavenging ability, whereas GQNPs containing an equivalent amount of Ga^3+^ significantly reduced ROS levels (Figure S2c). Previous studies have suggested that free Ga^3+^ may contribute to intracellular ROS generation and potentially increase oxidative stress [32, 34]. In GQNPs, however, the coordination of quercetin with Ga^3+^ apparently mitigates this potential pro‐oxidative effect, mainly owing to the potent ROS‐scavenging properties of quercetin.

### 2.2 Ferroptosis Attenuated by GQNPs

Before assessing the therapeutic effects of GQNPs, their cytotoxicity was first examined. SH‐SY5Y cells were incubated with GQNPs at concentrations of 4, 6, 8, 10, 15, and 20 µg/mL for 24 h, followed by cell viability measurement using the Cell Counting Kit‐8 (CCK‐8) assay. No significant cytotoxicity was observed at concentrations up to 20 µg/mL; therefore, 20 µg/mL was selected for subsequent experiments (Figure S3a). To investigate cellular uptake, inductively coupled plasma (ICP) analysis was carried out after SH‐SY5Y cells were incubated with GQNPs. The results showed substantial cellular uptake of GQNPs within 4 h (Figure S4a). Further confocal laser scanning microscopy (CLSM) analysis revealed a colocalization coefficient of 0.72 between GQNPs and lysosomes, suggesting that the nanoparticles predominantly accumulated in lysosomes after internalization (Figure S4b–d).

To explore the potential ferroptosis‐inhibitory effects of GQNPs in vitro, a PD cell model was established using 1‐methyl‐4‐phenylpyridinium (MPP^+^), a neurotoxin that selectively enters neurons via dopamine transporters and induces ATP depletion and excessive ROS generation [41]. Based on the CCK‐8 assay results (Figure S3b), SH‐SY5Y cells were treated with 4 mM MPP^+^ to induce ferroptosisrelated oxidative damage. Given the central role of oxidative stress in ferroptosis [42], the ROS‐regulating capability of GQNPs was compared with that of DFP, a clinically used iron chelator.

To evaluate ROS accumulation, intracellular ROS levels were measured using the DCFH‐DA probe. As shown in Figure 2a,c, MPP^+^ treatment significantly increased intracellular ROS levels, whereas GQNPs markedly suppressed ROS accumulation. In contrast, DFP showed only limited effects, suggesting that iron chelation alone was insufficient to effectively mitigate ferroptosis‐associated oxidative stress. Since mitochondria serve as the primary source of ROS generation [43], mitochondrial superoxide levels were further analyzed using the MitoSOX probe. The results indicate that MPP^+^ treatment significantly elevated mitochondrial superoxide levels, consistent with the trend observed for total ROS levels. However, GQNPs effectively reduced mitochondrial superoxide accumulation, whereas DFP showed limited effects (Figure 2b,c). These findings suggest that DFP has limited direct ROS‐scavenging capacity, thereby restricting its efficacy in suppressing oxidative stress‐associated ferroptotic injury [44]. In contrast, GQNPs not only scavenge ROS but also alleviate mitochondrial oxidative stress, resulting in remarkable effects on ferroptosis suppression in PD cells.

**FIGURE 2:**
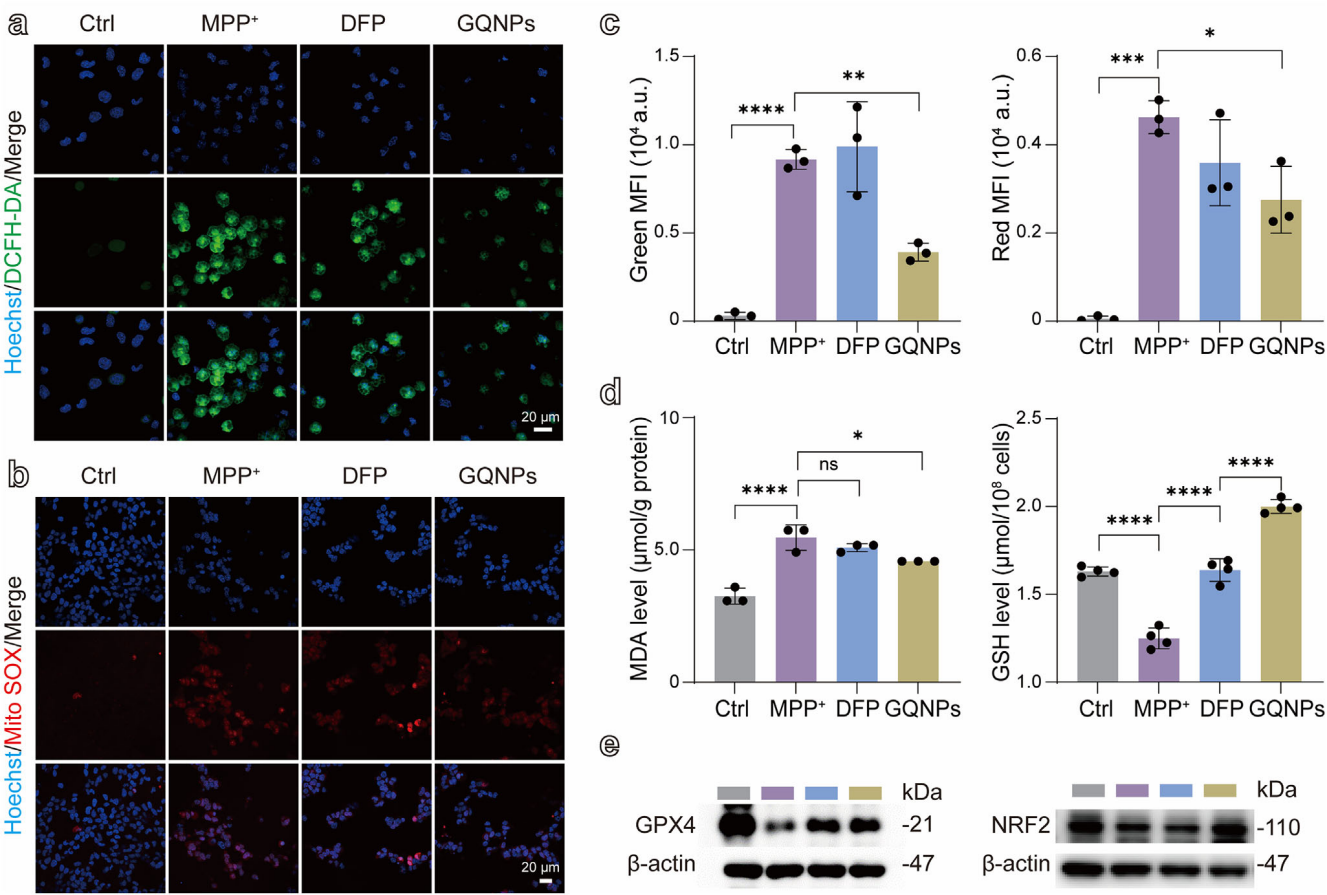
Ferroptosis inhibition by GQNPs in MPP^+^‐injured SH‐SY5Y cells. (a) Representative CLSM images of intracellular ROS levels detected using DCFH‐DA, (b) representative CLSM images of mitochondrial superoxide levels detected using MitoSOX, (c) quantification of mean fluorescence intensity in CLSM images, (d) MDA and GSH levels measured using a fluorescence microplate reader, (e) Western blot analysis of GPX4 and NRF2 protein expression across different treatment groups, with βactin used as the loading control. Scale bar: 20 µm.

To further assess the ferroptosis‐inhibitory effects of GQNPs, key biomarkers associated with lipid peroxidation, glutathione (GSH) metabolism, and GPX4 activity were examined. Malondialdehyde (MDA), a well‐established marker of lipid peroxidation [45], was measured to evaluate oxidative damage in PD cells following co‐incubation with GQNPs. As shown in Figure 2d, MPP^+^ treatment significantly increased intracellular MDA levels, indicating enhanced lipid peroxidation, whereas GQNPs effectively suppressed MDA accumulation. In contrast, DFP failed to significantly reduce MDA levels. These results highlight the superior ability of GQNPs to mitigate lipid peroxidation compared with conventional iron chelation.

Since ferroptosis is closely associated with GSH depletion, intracellular GSH levels were measured to assess the effects of GQNPs on cellular antioxidant defense. As shown in Figure 2d, MPP^+^ treatment caused a significant decline in GSH levels, indicating impaired antioxidant capacity. Although both DFP and GQNPs partially restored GSH levels, the increment caused by GQNPs was more pronounced. Since GSH serves as an essential cofactor for GPX4 [46], a key ferroptosis regulator, the expression of GPX4 was further examined by Western blot analysis. The results in Figure 2e and Figure S5a reveal that MPP^+^ treatment markedly downregulated GPX4 expression, whereas both DFP and GQNPs upregulated GPX4 expression to different extents, with GQNPs showing a more pronounced effect. This trend was consistent with the changes observed in GSH levels.

To further investigate the antioxidative mechanisms of GQNPs, the expression of NRF2, a key transcription factor regulating cellular redox homeostasis, was analyzed. Activated NRF2 binds to antioxidant response elements, leading to the upregulation of genes involved in oxidative stress resistance [47]. Western blot analysis showed that GQNPs significantly upregulated NRF2 expression, whereas DFP failed to restore NRF2 levels (Figure 2e and Figure S5b). These findings suggest that GQNPs not only replenish GSH and restore GPX4 expression but also enhance the overall antioxidant response through NRF2 regulation, providing a multi‐target approach for ferroptosis inhibition.

Collectively, these findings demonstrate that GQNPs effectively inhibit ferroptosis by reducing oxidative stress, suppressing lipid peroxidation, and reinforcing the cellular antioxidant defense system. Compared with DFP, which primarily functions through iron chelation, GQNPs exhibit superior ferroptosis‐inhibitory effects by simultaneously modulating oxidative stress, lipid peroxidation, and antioxidant signaling. These results highlight the potential of GQNPs as a promising multi‐target therapeutic strategy for ferroptosis inhibition in PD.

### 2.3 Molecular Mechanism of Ferroptosis Inhibition by GQNPs

Excessive iron accumulation is a significant contributor to PD pathology, as it elevates oxidative stress and promotes ferroptosis‐mediated neuronal loss. Iron overload facilitates dopamine oxidation, thereby promoting ROS generation. Additionally, excess iron activates the ubiquitin‐proteasome system and promotes GPX4 degradation, thereby accelerating ferroptotic cell death [48]. Given the chemical similarity between Ga^3+^ and Fe^3+^, Ga^3+^ can bind to transferrin [49]. It is therefore reasonable to hypothesize that Ga^3+^ may regulate Fe^3+^ uptake and thereby inhibit ferroptosis in PD. To verify this hypothesis, MPP^+^‐treated SH‐SY5Y cells were incubated with gallium nitrate. The decreased intracellular iron level demonstrated that Ga^3+^ treatment significantly reduced total iron levels, suggesting its potential to mitigate ferroptosis by modulating iron metabolism (Figure S6).

To further assess the effects of GQNPs on iron homeostasis, their efficacy was compared with that of DFP. As shown in Figure 3a, MPP^+^ treatment markedly increased iron accumulation in PD cells, while treatment with either GQNPs or DFP effectively reduced intracellular iron levels. Given that Fe^2+^ is a key driver of ferroptosis, intracellular Fe^2+^ levels were further analyzed using a fluorescent Fe^2+^ probe. The results revealed that both GQNPs and DFP effectively reduced Fe^2+^ accumulation in PD cells (Figure 3b,c). Notably, GQNPs exhibited efficacy comparable to that of DFP in reducing both total iron and Fe^2+^ levels, suggesting that GQNPs achieved a similar degree of iron reduction to DFP, which may contribute to ferroptosis inhibition.

**FIGURE 3:**
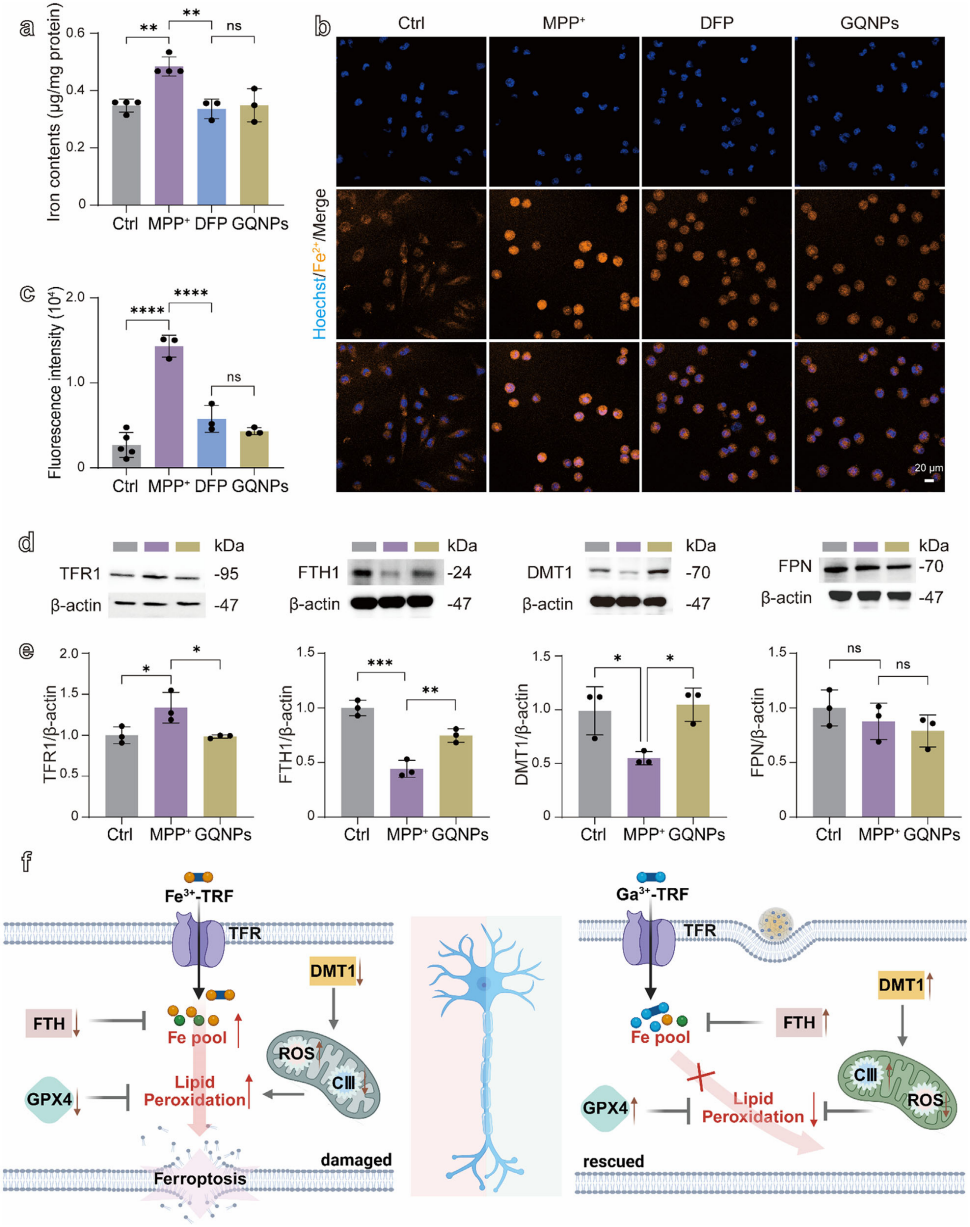
Regulation of iron content and iron‐metabolism‐related proteins by GQNPs in PD cells. (a) Quantification of total iron content in SH‐SY5Y cells following different treatments, (b) CLSM images of Fe^2+^ staining in SH‐SY5Y cells, (c) quantitative analysis of Fe^2+^ fluorescence intensity from panel b, (d) representative Western blot images showing the expression of key iron metabolism‐related proteins: TFR1, FTH1, DMT1, and FPN, as well as (e) quantitative analysis of protein expression levels normalized to β‐actin, (f) schematic representation of the proposed mechanism by which GQNPs alleviate ferroptosis in PD cells. Scale bar: 20 µm.

To further dissect the respective contributions of Ga^3+^ and quercetin to the multi‐target ferroptosis‐inhibitory effect of GQNPs, additional comparative experiments were performed in the MPP^+^‐induced PD cell model using Ga^3+^, quercetin, their physical mixture, and GQNPs. For oxidative stress‐related indices, including ROS, GSH, and MDA, the quercetin‐treated group exhibited stronger protective effects than the Ga^3+^‐treated group, whereas GQNPs showed significantly improved antioxidant effects compared with Ga^3+^ alone (Figure S7a–d). These findings suggest that the antioxidant activity of GQNPs was mainly attributable to the quercetin component. In contrast, Ga^3+^‐containing treatments were more effective than quercetin alone in reducing intracellular iron levels, indicating that Ga^3+^ played a distinct role in regulating cellular iron homeostasis (Figure S7e). Taken together, these data support the complementary functions of the two components in GQNPs, with quercetin mainly responsible for antioxidant protection and Ga^3+^ contributing more prominently to iron regulation. Notably, although the physical mixture showed effects comparable to those of GQNPs in vitro, it was prone to precipitation and exhibited poor stability. In contrast, GQNPs displayed improved physicochemical stability, supporting their greater potential for further biological and translational applications.

To further elucidate the mechanisms by which GQNPs regulated iron accumulation, the expression of key iron‐metabolism‐related proteins was examined. Cellular iron uptake is primarily mediated by TFR1, which allows the internalization of transferrin‐bound Fe^3+^. Once inside the endosome, Fe^3+^ is reduced to Fe^2+^ by six‐transmembrane epithelial antigen of the prostate 3 and subsequently transported to the cytoplasm or organelles by divalent metal transporter 1 (DMT1) [50, 51]. Iron ions that are not immediately utilized are either stored in ferritin or exported via ferroportin (FPN) [52]. To further explore how GQNPs influenced iron metabolism, the expression levels of TFR1, DMT1, ferritin heavy chain 1 (FTH1), and FPN were evaluated.

Western blot analysis showed that TFR1 expression was significantly upregulated in MPP^+^‐treated SH‐SY5Y cells, which may contribute to excessive iron uptake. Treatment with GQNPs significantly downregulated TFR1 expression, thereby reducing iron influx. Additionally, FTH1 expression was significantly elevated following GQNPs treatment, suggesting enhanced cellular iron storage capacity (Figure 3d,e). Given that quercetin, a key component of GQNPs, has been reported to regulate ferritin stability and iron storage, the upregulation of FTH1 may further contribute to ferroptosis inhibition.

DMT1 functions as a divalent metal transporter and plays an important role in intracellular Fe^2+^ transport. In this study, MPP^+^ treatment decreased DMT1 expression, which differs from the conventional expectation that pathological iron accumulation is often accompanied by increased iron uptake. Notably, GQNPs treatment restored DMT1 expression, suggesting a potential role in re‐establishing iron homeostasis (Figure 3d,e). Since DMT1 transports multiple divalent metal ions, including Fe^2+^, Ca^2+^, and Mn^2+^, its expression level may not directly correlate with total intracellular iron levels. Instead, DMT1 may also be involved in intracellular iron redistribution and mitochondrial iron handling [53]. Consistent with this possibility, mitochondrial ferrous iron staining revealed elevated mitochondrial Fe^2+^ levels in MPP^+^‐treated cells, whereas GQNPs treatment significantly reduced mitochondrial iron accumulation (Figure S8). In parallel, the expression of mitochondrial respiratory chain complex III was markedly decreased after MPP^+^ treatment and was effectively restored by GQNPs (Figure S9), further supporting the association between improved iron distribution and mitochondrial function. It is also possible that the reduction of DMT1 expression observed in the model group represents a compensatory cellular response to iron overload. Nevertheless, in the context of this study, changes in DMT1 expression appear to be more closely associated with the normalization of intracellular iron distribution and mitochondrial function than with a direct linear relationship with total iron uptake.

Further analysis of FPN expression revealed no significant variation among the treatment groups, suggesting that these treatments did not markedly alter the iron export pathway (Figure 3d,e). Collectively, these findings demonstrate that GQNPs restored iron homeostasis in MPP^+^‐treated cells by reducing TFR1‐mediated iron influx, enhancing FTH1‐associated iron storage, and improving mitochondrial iron distribution. Meanwhile, the restoration of DMT1 in the GQNPs‐treated group may be more closely related to mitochondrial functional recovery rather than to a simple increase in iron uptake (Figure 3f). By limiting iron influx, enhancing iron storage, and restoring mitochondrial function, GQNPs exhibited a multifaceted role in ferroptosis inhibition, highlighting their potential as a promising multitarget therapeutic strategy for PD treatment.

### 2.4 Treatment of MPTP‐Induced PD Mice by GQNPs

To evaluate the therapeutic efficacy of GQNPs in vivo, the 1‐methyl‐4‐phenyl‐1,2,3,6‐tetrahydropyridine (MPTP)‐induced PD mouse model was established. MPTP is a well‐established neurotoxin that selectively induces dopaminergic neuronal degeneration and recapitulates key pathological and behavioral features of PD, including motor and cognitive dysfunction [54]. Based on previous reports with minor modifications, a subacute PD model was established by intraperitoneal administration of MPTP at 35 mg/kg once daily for 8 consecutive days [55, 56].

A major challenge in drug delivery to the central nervous system (CNS) is the BBB, which restricts the brain accumulation of many therapeutic agents. Various strategies have been explored to enhance CNS drug delivery, including ligand‐mediated transcytosis [57], BBB‐penetration enhancers [58], and focused ultrasound techniques [59]. Although significant progress has been made, these strategies may still face limitations in convenience, safety, or clinical practicality for CNS disease treatment [60]. Recently, intranasal administration has emerged as a non‐invasive and facile route for brain‐targeted drug delivery by leveraging the physiological pathway between the nasal cavity and the CNS [60, 61]. Given its safety, efficiency, and brain‐targeting properties, intranasal delivery was selected for GQNPs administration in this study.

To confirm whether GQNPs could be effectively delivered into the brain after intranasal administration, the intrinsic fluorescence emission of GQNPs at approximately 600 nm was used for in vivo tracking with an IVIS imaging system (Figure S10). Mice were intranasally administered with GQNPs, and brain tissues were collected at different time points post‐administration for imaging analysis. The results showed that the fluorescence signal peaked at 2 h post‐administration, followed by a gradual decline over time. The fluorescence signal was nearly undetectable at 72 h, suggesting that GQNPs were largely cleared from the brain, which was further supported by quantitative analysis of gallium content (Figure 4a,b). These biodistribution results indicate that GQNPs can be effectively delivered into the brain via intranasal administration.

**FIGURE 4:**
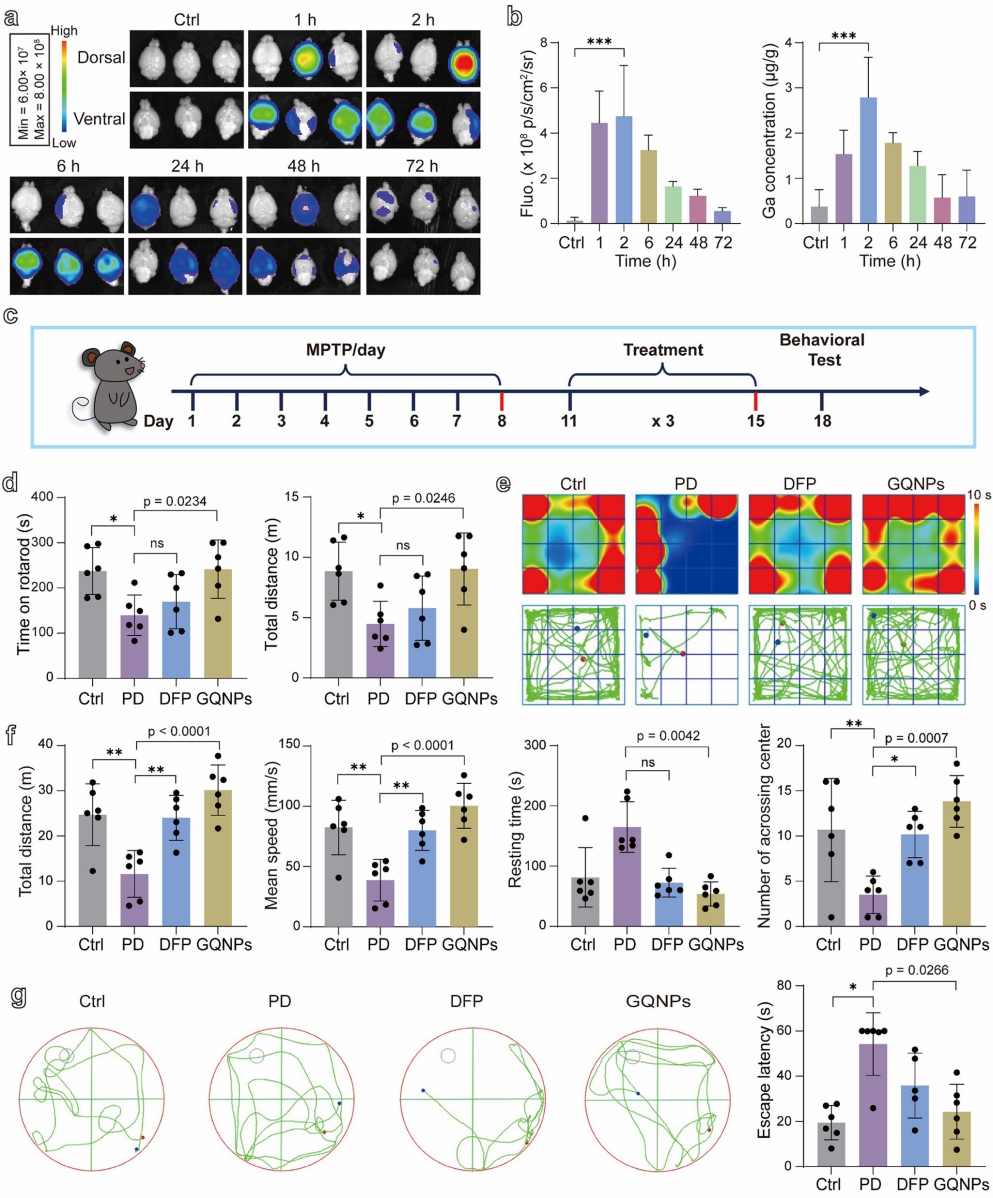
Therapeutic effects of GQNPs in MPTP‐induced PD mice. (a) Fluorescence images of excised mouse brains at different time points following intranasal administration, showing dorsal and ventral brain tissue, (b) quantification of brain fluorescence intensity over time and gallium content in brain tissue determined by ICP, n = 3, (c) experimental timeline depicting MPTP‐induced PD model establishment, treatment administration, and behavioral assessments, (d) rotarod test evaluating motor coordination, n = 6, (e) open field test showing representative movement trajectories of mice in different groups, and (f) quantitative analyses of open field test parameters, n = 6, (g) Morris water maze test assessing spatial learning and memory, n ≥ 5.

To ensure comparability among experimental groups, MPTP‐induced PD mice were pre‐screened and stratified according to rotarod performance (Figure S11). The mice were then divided into four groups: control group (healthy C57BL/6 mice), PD group (MPTP‐induced PD mice), DFP‐treated group (PD mice treated with DFP intranasally), and GQNPs‐treated group (PD mice treated with GQNPs intranasally). Each group of mice was subjected to behavioral experiments after three intranasal treatments (Figure 4c).

The rotarod test was used to evaluate balance and motor coordination of mice [62]. Compared with the control group (237.6 s), PD mice exhibited a significant decline in rotarod performance (139.6 s, *p* < 0.05), indicating impaired motor coordination. Although DFP‐treated mice showed a modest improvement (170.0 s), the difference was not statistically significant compared with the PD group. In contrast, GQNPs‐treated mice showed a significant improvement (241.4 s, *p* = 0.0234), with motor coordination restored to near‐control levels. Similar trends were observed in the total distance traveled, further supporting the superior efficacy of GQNPs in enhancing motor function (Figure 4d).

To further assess spontaneous locomotion and exploratory behavior, mice underwent the open field test. Heatmap analysis revealed that PD mice preferentially stayed in peripheral areas, indicating reduced exploratory behavior. Both DFP and GQNPs treatment increased exploratory behaviors, as reflected by a broader movement area (Figure 4e and Figure S12). The total distance traveled was significantly lower in PD mice (11.6 m) than in healthy mice (24.7 m), whereas DFP (24.0 m, *p* < 0.01) and GQNPs treatment (30.1 m, *p* < 0.0001) significantly restored locomotor activity. Additional analysis of mean speed and resting time further confirmed that GQNPs treatment was more effective than DFP in restoring locomotor activity. Furthermore, GQNPs‐treated mice showed a significantly higher number of crossings into the central area, suggesting reduced anxiety‐like behavior and enhanced exploratory activity (Figure 4f). Collectively, these results indicate that GQNPs effectively improved motor function and exploratory behavior in PD mice, outperforming DFP treatment.

To evaluate the effects of GQNPs on cognitive function, the Morris water maze test was conducted [63]. This test assesses spatial learning and memory by measuring the latency to locate a hidden escape platform. Compared with the control group, PD mice exhibited significantly prolonged escape latency, indicating cognitive deficits (Figure 4g and Figure S13). Although DFP treatment did not significantly reduce escape latency, GQNPs‐treated mice showed marked improvement and navigated the maze more efficiently than untreated PD mice. The number of platform crossings was also quantified. Compared with the model group, GQNPs‐treated mice exhibited an increased number of platform crossings (Figure S14a), indicating improved spatial memory. These results suggest that GQNPs effectively alleviated cognitive impairments in PD mice.

Importantly, no significant differences were observed among the groups in swimming distance, mean velocity, or immobility time in the Morris water maze (Figure S14b–d), indicating comparable motor performance across groups. Therefore, the observed differences in escape latency and platform crossings were unlikely to be caused by differences in locomotor ability and could be mainly attributed to cognitive function. In addition, the rotarod test evaluates motor coordination associated with the basal ganglia and cerebellum, with the substantia nigra being a key region affected in PD pathology [64]. In contrast, the Morris water maze primarily assesses hippocampus‐dependent cognitive function [65]. Thus, the combined use of these behavioral tests enabled independent evaluation of motor and cognitive deficits in the PD model.

In brief, the above results demonstrate that GQNPs significantly improved both motor and cognitive functions in MPTP‐induced PD mice. Compared with DFP, which primarily acts as an iron chelator, GQNPs exhibited superior therapeutic efficacy by integrating multi‐target ferroptosis inhibition with efficient intranasal brain delivery.

### 2.5 Pathological Changes of Treated Mice

In addition to behavioral assessments, the therapeutic effects of GQNPs were further evaluated by examining pathological changes in MPTP‐induced PD mice. Histological and biochemical markers associated with dopaminergic neurodegeneration and ferroptosis were analyzed to assess neuronal damage and recovery. Nissl bodies play a critical role in neuronal protein synthesis and serve as indicators of neuronal health and integrity [66]. As shown in Figure 5a, the hippocampal region of PD mice exhibited a substantial reduction in Nissl bodies, reflecting neuronal damage and degeneration. In contrast, GQNPs treatment increased the number of Nissl bodies, suggesting a therapeutic effect on neuronal structure. To further evaluate dopaminergic neuroprotection, tyrosine hydroxylase (TH), the rate‐limiting enzyme in catecholamine biosynthesis, was examined by immunofluorescence [67]. Compared to PD mice, which exhibited an apparent reduction in TH‐positive signals, GQNPs‐treated mice showed partially restored TH levels, indicating improved dopaminergic neuron integrity (Figure 5a).

**FIGURE 5:**
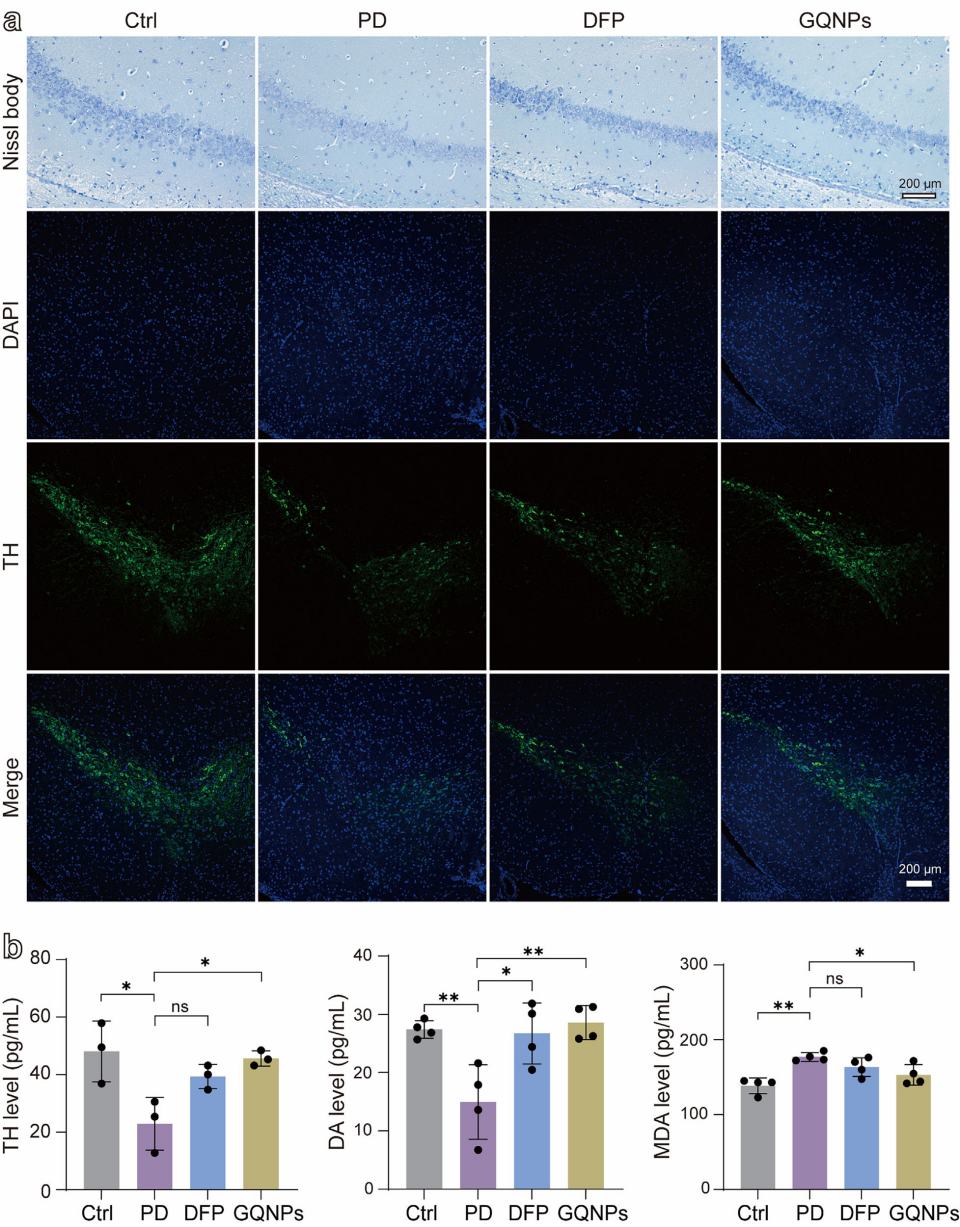
Pathological improvement in PD mice after GQNPs treatment. (a) Representative images of Nissl staining and TH immunofluorescence staining in the substantia nigra across different treatment groups; cell nuclei were stained with DAPI, and (b) ELISA quantification of key pathological biomarkers in the midbrain of mice, including TH level (n = 3), DA concentration (n = 4), and MDA level (n = 4). Scale bar: 200 µm.

To quantitatively assess dopaminergic function, TH and DA levels in substantia nigra tissues were measured using enzyme‐linked immunosorbent assay (ELISA). The results revealed that TH levels were significantly reduced in PD mice but were partially restored following GQNPs treatment. Similarly, DA level, which was significantly depleted in PD mice, was notably increased in the GQNPs‐treated group (Figure 5b). These results support the dopaminergic neuroprotective effect of GQNPs, as reflected by improved TH expression and DA production. To investigate whether the neuroprotective effects of GQNPs were associated with ferroptosis inhibition, MDA, a widely recognized biomarker of ferroptosis, was quantitatively analyzed. Following treatment with GQNPs, the elevated MDA level in PD mice was significantly reduced, suggesting that GQNPs effectively mitigated ferroptosis‐induced lipid peroxidation (Figure 5b). These results indicate that GQNPs alleviated neuronal damage not only by restoring dopaminergic function but also by suppressing ferroptosis‐associated lipid peroxidation.

### 2.6 Biosafety Evaluation of GQNPs

Hematoxylin‐eosin (H&E) staining of major tissues and organs, including the cerebral cortex, midbrain, heart, liver, spleen, lung, and kidney, showed no obvious histopathological abnormalities after intranasal administration of GQNPs (Figure 6a and Figure S15). Furthermore, routine blood parameters, including white blood cell (WBC), red blood cell (RBC), hemoglobin (HGB), and platelet (PLT) as well as biochemical indices, including alanine aminotransferase (ALT), albumin (ALB), creatinine (CREA), and total protein (TP), showed no significant differences between GQNPs‐treated mice and normal controls (Figure 6b,c). These results indicate that GQNPs exhibited favorable preliminary biosafety and biocompatibility. Moreover, the doses of each component used in the animal experiments were within a safe range, supporting their suitability for further therapeutic exploration [68, 69, 70].

**FIGURE 6:**
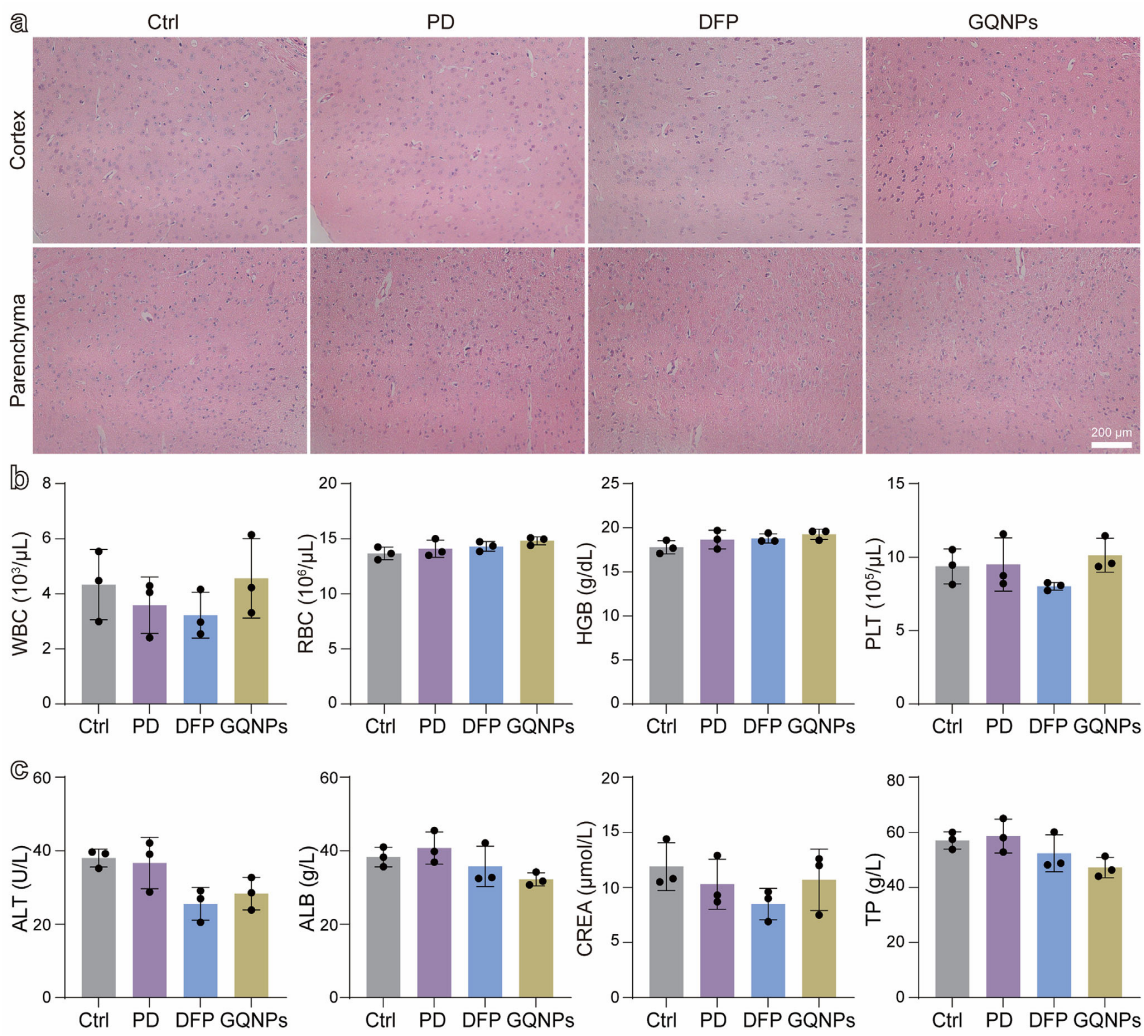
Biocompatibility assessment of GQNPs in vivo. (a) Representative H&E staining images of the cerebral cortex and parenchyma from different treatment groups, (b) routine blood test results, including WBC, RBC, HGB, and PLT counts (n = 3), and (c) blood biochemical results, including ALT, ALB, CREA, and TP levels (n = 3). Scale bar: 200 µm.

## 3 Discussion

The rational design of GQNPs integrates iron metabolism regulation, iron chelation, and antioxidant activity into a unified nanoplatform. Compared with conventional ferroptosis‐targeting strategies, this approach provides a more comprehensive framework for addressing the multifactorial nature of ferroptosis in PD. Iron chelators such as DFP and deferoxamine can reduce pathological iron accumulation; however, their therapeutic efficacy may be limited when ferroptosis is driven by multiple interconnected pathological events. As ferroptosis involves a cascade of interconnected processes, including iron dysregulation, lipid peroxidation, and oxidative stress, targeting iron availability alone is often insufficient. This limitation has prompted the development of strategies that combine iron chelators with antioxidant intervention [71], highlighting the need for multi‐target ferroptosis regulation. Emerging nanoplatforms, such as gallic acid–selenium nanoparticles [72], double–selenium nanospheres [73], and Icariside II–Fe^3^^+^ nanozymes [74], have demonstrated the feasibility of modulating multiple ferroptosis‐related pathways. However, many of these systems are still mainly centered on redox regulation. In contrast, our design strategy emphasizes the simultaneous modulation of iron metabolism and oxidative stress, aiming to interrupt ferroptosis at multiple critical nodes.

The incorporation of Ga^3+^ provides a distinct iron‐mimetic strategy for regulating iron metabolism by interfering with transferrin‐associated iron transport. This strategy has been widely explored in antibacterial and anticancer contexts but remains less investigated in neurodegenerative diseases. Notably, the inherent limitations of free gallium, such as hydrolysis‐related low bioavailability [75], and the lack of intrinsic antioxidant activity [49], may be addressed through coordination with quercetin. As a polyphenolic ligand, quercetin contributes antioxidant activity and mild iron‐chelating capacity, enabling functional complementarity within the nanoparticle system. This ligand‐oriented design strategy, which integrates complementary biological functions, represents an underexplored direction in metal–polyphenol nanomaterials for neurodegenerative diseases [76].

Rather than replacing existing antioxidant or iron‐regulation strategies, GQNPs integrate their respective advantages into a single system. By leveraging the chemical similarity between Ga^3+^ and Fe^3+^ in iron‐associated transport processes, GQNPs provide a complementary approach to ferroptosis inhibition. Mechanistically, Ga^3+^ interferes with transferrin‐mediated iron uptake, thereby reducing intracellular iron accumulation. This effect was further supported by the downregulation of TFR1 and the upregulation of FTH1, which collectively contributed to improved iron sequestration and homeostasis. Through this coordinated regulation, GQNPs effectively attenuated lipid peroxidation and oxidative stress, thereby disrupting the pathological cycle associated with ferroptosis‐driven neurodegeneration.

In addition to iron regulation, GQNPs also influenced mitochondrial function. The restoration of DMT1 expression and mitochondrial respiratory complex III suggested an association between normalized iron distribution and improved mitochondrial integrity. Although markers such as ROS accumulation and mitochondrial dysfunction may overlap with other forms of cell death, the combined modulation of iron levels, ferroptosis‐related proteins, lipid peroxidation, and the GPX4 antioxidant system supports ferroptosis as the primary pathway targeted by GQNPs.

Furthermore, intranasal administration enabled efficient delivery of GQNPs to the CNS, allowing GQNPs to exert therapeutic effects in vivo. The observed improvement in both motor and cognitive functions, together with favorable preliminary biosafety results, supports the potential of this strategy. Overall, by integrating iron regulation, oxidative stress suppression, and mitochondrial protection, GQNPs represent a cooperative multi‐target approach for ferroptosis inhibition in PD.

Despite the promising therapeutic potential of GQNPs demonstrated in the MPTP‐induced PD model, several limitations should be acknowledged. First, although this study provides initial evidence for multi‐target ferroptosis inhibition and short‐term biosafety, validation in additional PD models is required to establish broader applicability across different pathological contexts. Second, systematic optimization of the dosing regimen is needed. Future dose–response studies will be essential for determining the optimal concentration and administration frequency to maximize therapeutic efficacy while minimizing potential side effects. Third, comprehensive long‐term safety evaluation remains necessary. In particular, the biodistribution, metabolism, and potential accumulation of gallium ions in vivo should be carefully assessed to ensure safety during prolonged use. Finally, although DFP was used as a clinically relevant iron chelator for comparison, future studies incorporating additional ferroptosis‐targeting agents, such as deferoxamine, ferrostatin‐1, and liproxstatin‐1, as well as established PD therapeutics such as levodopa, would provide a more comprehensive evaluation of therapeutic performance. Notably, differences in BBB permeability and mechanisms of action among these agents may influence their therapeutic applicability [77]. Addressing these limitations will be critical for advancing GQNPs toward further preclinical development for PD and other ferroptosis‐related neurodegenerative diseases.

## 4 Conclusions

In this study, intranasally administered GQNPs attenuated ferroptosis‐associated damage in MPP^+^‐injured SH‐SY5Y cells and MPTP‐induced PD mice through coordinated regulation of iron homeostasis, oxidative stress, lipid peroxidation, and mitochondrial function. GQNPs reduced iron accumulation, restored ferroptosis‐related antioxidant defenses, improved motor and cognitive performance, and showed no evident short‐term toxicity under the tested conditions. This study is limited by the use of a single neurotoxin‐based animal model, the absence of systematic dose optimization, and the lack of long‐term safety assessments. Validation in chronic and α‐synuclein‐related PD models, together with optimized dosing and long‐term safety studies, is therefore required before clinical translation. These findings support GQNPs as a potential multi‐target nanoplatform for further preclinical investigation of ferroptosis‐associated neurodegenerative diseases.

## 5 Materials and Methods

### 5.1 Materials

Quercetin was purchased from Suzhou Utepo Technology Co., Ltd. Polyvinylpyrrolidone (PVP, Mw ≈ 40 kDa) was obtained from Solarbio. Gallium nitrate (Ga(NO_3_)_3_·9H_2_O) was supplied by Jiangsu A.K.X. Material Technology Co., Ltd. A dialysis membrane with a molecular weight cut‐off (MWCO) of 100 kDa was obtained from Suzhou Suke Lean Instrument Co., Ltd. 1‐Methyl‐4‐phenylpyridinium iodide (MPP^+^ iodide) was purchased from Suzhou Utepo Technology Co., Ltd., and MPTP hydrochloride was obtained from Beyotime Biotechnology. Deferiprone (DFP) was purchased from TargetMol Chemicals Inc. Milli‐Q ultrapure water (18 MΩ·cm) was used in all experiments. All chemicals and reagents were used as received without further purification.

### 5.2 Synthesis of GQNPs

GQNPs were synthesized using a coordination self‐assembly method. Briefly, 100 mg of PVP was dissolved in 3 mL of methanol under continuous stirring. Subsequently, 8 mg of quercetin, dissolved in 1.5 mL of methanol, was added dropwise to the PVP solution under stirring. After stirring for 1 h, 40 mg of Ga(NO_3_)_3_·9H_2_O, dissolved in 4 mL of methanol, was gradually introduced into the mixture and allowed to react under continuous stirring for 6 h. The resulting solution was dialyzed against water for 18 h to remove excess methanol and uncoordinated metal ions. All the above steps were carried out at room temperature. The gallium concentration in the final GQNPs dispersion was determined using inductively coupled plasma‐optical emission spectrometry (ICP‐OES, Thermo Fisher, iCAP 7000).

### 5.3 Characterization of GQNPs

UV–vis absorption spectra were recorded using a PerkinElmer LAMBDA 750 spectrophotometer. Fluorescence emission spectra were obtained using an Edinburgh fluorescence spectrophotometer (FLS980). TEM images were acquired using an FEI Tecnai G2 microscope operating at an accelerating voltage of 120 kV. The hydrodynamic size of GQNPs was measured at 25°C using a Malvern Zetasizer Nano ZS90 equipped with a solid‐state He–Ne laser (λ = 633 nm).

### 5.4 Total Antioxidant Capability Assay of GQNPs

The total antioxidant capability of GQNPs was evaluated using a Total Antioxidant Capacity (T‐AOC) assay kit (Cat. No. S0121, Beyotime). In brief, 20 µL of peroxidase working solution was added to each well of a 96‐well plate. Various concentrations of Ga^3+^ (0.5, 1, 3, and 5 µg/mL) or GQNPs at equivalent gallium concentrations were added at 10 µL per well. Subsequently, 170 µL of ABTS working solution was added to each well. After incubation at room temperature for 6 min, the absorbance at 414 nm was measured using a microplate reader (PerkinElmer EnSpire, Singapore).

### 5.5 Cell Culture

SH‐SY5Y cells were cultured in Dulbecco's Modified Eagle Medium/Nutrient Mixture F‐12 (DMEM/F12) supplemented with 10% FBS and 1% antibiotics (penicillin and streptomycin). The cells were maintained in a humidified incubator at 37°C with 5% CO_2_ under standard cell culture conditions.

### 5.6 Cell Viability Analysis

SH‐SY5Y cells were seeded into 96‐well plates at a density of 5 × 10^3^ cells per well and incubated for 24 h. Cells were then treated with various concentrations of GQNPs (0, 4, 6, 8, 10, 15, 20 µg/mL) or MPP^+^ (0, 1, 2, 4, 6, 8, 10 mM) for another 24 h. After treatment, the culture medium was replaced with 200 µL of fresh medium containing 10% CCK‐8 reagent (Cat. No. C0043, Beyotime), and the cells were incubated for an additional 1 h at 37°C. The absorbance at 450 nm was measured using a microplate reader (PerkinElmer EnSpire, Singapore). Cell viability was calculated using the following formula: Cell viability (%) = (A__sample_ − A__blank_)/(A__control_ − A__blank_) ×100%, where A__sample_ is the absorbance of the treated cells, A__blank_ is the absorbance of the blank control group, and A__control_ is the absorbance of the untreated control cells.

### 5.7 Cellular Uptake Analysis

SH‐SY5Y cells were seeded into confocal dishes at a density of 1 × 10^6^ cells per well. After cell attachment, GQNPs were added at a concentration of 20 µg/mL of was and incubated with the cells for 0, 2, and 4 h. After incubation, the cells were washed three times with PBS to remove excess nanoparticles. To visualize cellular uptake, lysosomal fluorescent dye (Cat. No. C1047S, Beyotime) was added at a concentration of 50 nM and incubated at 37°C for 60 min. Nuclei were counterstained with Hoechst 33342 at 37°C for 25 min. Fluorescence images were captured using CLSM (FV1200, Olympus, Japan; excitation: 488/488/405 nm, emission: 600/511/460 nm).

### 5.8 ROS Fluorescence Imaging

SH‐SY5Y cells were seeded into 35 mm glass‐bottom dishes at a density of 5 × 10^4^ cells per dish and cultured for 24 h. The cells were pre‐incubated with 20 µg/mL GQNPs or 10 µM DFP for 4 h, followed by exposure to fresh medium containing 4 mM MPP^+^ for an additional 24 h. After treatment, intracellular ROS levels were detected using DCFH‐DA (Cat. No. C0033S, Beyotime), and mitochondrial ROS levels were assessed using MitoSOX (Cat. No. M36007, Invitrogen). The cells were stained with DCFH‐DA for 30 min and with MitoSOX for 10 min at 37°C, followed by nuclear staining with Hoechst 33342 (Cat. No. C1017, Beyotime). Fluorescence imaging was performed using CLSM (FV1200, Olympus, Japan) with excitation wavelengths of 488/560/405 nm and emission wavelengths of 525/610/460 nm, respectively. The average fluorescence intensities were quantified using ImageJ software.

### 5.9 Measurement of MDA and GSH Levels

MDA and GSH levels were quantified according to the manufacturer's instructions. Briefly, SH‐SY5Y cells were seeded at a density of 5 × 10^6^ cells per flask and cultured for at least 24 h. After treatment as described above, the cells were lysed using cell lysis buffer. To ensure complete cell lysis, ultrasonic disruption was performed. All steps were conducted on ice to preserve sample integrity. The cellular MDA and GSH levels were calculated using the following formulas: MDA (µmol/mg protein) = MDA concentration/protein content (Cat. No. S0131S, Beyotime); GSH (µmol/10^4^ cell) = 1.033 × ((A__sample_ − A__control_) − 0.006)/cell number (Cat. No. G0206W, Geruisi‐bio).

### 5.10 Measurement of Cellular Iron Content

The total iron content in SH‐SY5Y cells was measured using a Cell Iron Assay Kit (Cat. No. BC5315, Solarbio). Cells were seeded in culture flasks at a density of 3 × 10^6^ to 5 × 10^6^ cells per flask and incubated for at least 24 h. After treatment, the cells were washed with PBS, collected, and lysed using cell lysis buffer. The lysates were centrifuged at 12 000 rpm for 10 min, and the supernatants were collected for further analysis. For protein quantification, 20 µL of each sample was added to a 96‐well plate, followed by an equal volume of BCA working solution (Cat. No. P0010, Beyotime). The plate was incubated at 37°C for 40 min. For iron quantification, another 20 µL aliquot of each sample was added to a separate 96‐well plate, followed by the iron content detection working solution. The plate was incubated at room temperature for 10 min. The absorbance at 450 nm and 510 nm was measured using a microplate reader. The iron content was calculated using the following formulas: cellular iron content (ng/mg protein) = 27922.5 × (A__sample_ − A__blank_)/(A__standard_ − A__blank_)/protein concentration; cell iron content (ng/10^4^ cells) = 27.922 × (A__sample_ − A__blank_)/(A__standard_ − A__blank_).

### 5.11 Intracellular Ferrous Ion Detection

The intracellular Fe^2+^ content was measured using an FeRhoNox‐1 probe‐based assay kit (Cat. No. MX4558, MKBio). SH‐SY5Y cells were seeded into 35 mm glass‐bottom dishes at a density of 5 × 10^4^ cells per dish and cultured for 24 h. The cells were pre‐incubated with 20 µg/mL GQNPs or 10 µM DFP for 4 h, followed by treatment with 4 mM MPP^+^ for an additional 24 h. Following incubation, the cells were washed three times with PBS and stained with FeRhoNox‐1 (5 µM) for 60 min. After staining, the cells were washed twice with PBS and counterstained with Hoechst 33342 (Cat. No. C1017, Beyotime) for 30 min at 37°C. Fluorescence images were acquired using CLSM (FV1200, Olympus, Japan) with excitation wavelengths of 559 and 405 nm and emission wavelengths of 575 and 460 nm, respectively. The fluorescence intensities were quantified using ImageJ software.

### 5.12 Western Blotting Analysis

For protein expression analysis, SH‐SY5Y cells were treated as described above and then lysed using radioimmunoprecipitation assay (RIPA) buffer supplemented with protease inhibitors. The cells were lysed on ice, and the lysates were centrifuged at 12 000 rpm for 10 min at 4°C. Protein concentrations were determined using a BCA Protein Assay Kit (Cat. No. P0010, Beyotime). Proteins (20 µg per sample) were separated by SDS‐PAGE and transferred onto polyvinylidene difluoride (PVDF) membranes. The membranes were blocked with protein‐free rapid blocking buffer (Epizyme, PS108P) for 20 min and then incubated overnight at 4°C with primary antibodies against DMT1 (1:2000, Cat. No. 20507‐1‐AP, Proteintech), FTH1 (1:2000, Cat. No. 11682‐1‐AP, Proteintech), TFR1 (1:2500, Cat. No. 46222, Cell Signaling Technology), FPN (1:2000, Cat. No. ab239511, Abcam), GPX4 (1:2000, Cat. No. 30388‐1‐AP, Proteintech), Nrf2 (1:1500, Cat. No. 16396‐1‐AP, Proteintech), β‐actin (1:2000, Cat. No. BM3873), GAPDH (1:2000, Cat. No. 60004‐1, Proteintech). After washing four times with 1× TBST, the membranes were incubated with HRP‐conjugated secondary antibodies for 1.5 h at room temperature. Protein bands were visualized using an FDbio‐Pico ECL Kit (Cat. No. FD8000, FDbio Science) and detected using a FluorChem M Imaging System (ProteinSimple, San Jose, CA). The relative gray values of the protein bands were quantified using ImageJ software.

### 5.13 Detection of Mitochondrial Complex Activity

SH‐SY5Y cells were treated as described in the previous sections and then collected for mitochondrial complex activity assessment. According to the assay protocol, the cells were resuspended in extraction buffer and homogenized using a mortar. The homogenate was first centrifuged at 600 × *g* for 10 min at 4°C to remove cell debris. The supernatant was then transferred to a new centrifuge tube and centrifuged at 18 000 *g* for 15 min at 4°C to pellet the mitochondria. The mitochondrial pellet was resuspended in 200 µL of extraction buffer, and the mitochondria were further disrupted using an ultrasonic homogenizer (20% power, 5 s pulses and 10 s intervals, repeated 15 times). The subsequent steps followed the manufacturer's instructions for the assay kits. The activity of mitochondrial respiratory chain complex III was determined by measuring the absorbance at 550 nm using a microplate reader (Cat. No. BC3245, Solarbio).

### 5.14 Fluorescence Imaging In Vivo

Specific‐pathogen‐free (SPF) C57BL/6 mice (male, 12–15 weeks old) were provided by the Animal Center of Soochow University. For intranasal administration, mice were anesthetized with 1.5% isoflurane and placed in a supine position. A microcapillary tube with an outer diameter of 0.25 mm was connected to a syringe containing GQNPs, and the free end was gently inserted into the nasal cavity of each mouse. A total volume of 20 µL of GQNPs, with a gallium concentration of 5 mg/mL, was delivered unilaterally into the nasal passage. To assess the brain accumulation and biodistribution of GQNPs, mice were euthanized at 1, 2, 6, 24, 48, and 72 h post‐administration (n = 3 per group). Brain tissues were harvested for IVIS imaging (PerkinElmer), and fluorescence signals were analyzed using Living Image 4.2 software with spectral unmixing algorithms. After imaging, brain tissues were digested with an acidic digestion solution, and gallium concentrations at different time points were quantified using ICP.

### 5.15 Establishment and Treatment of PD Mouse Model

Male C57BL/6 mice aged 12–18 weeks were acclimated for 7 days before experiments. Because male mice are more sensitive to MPTP‐induced neurotoxicity, only male mice were used for model establishment in this study. The PD model was induced in C57BL/6 mice by intraperitoneal injection of MPTP (35 mg/kg) once daily for 8 consecutive days according to previous reports with modifications. This dosage has been reported to be sufficient to induce PD‐like behavioral phenotypes in mice [55, 56]. The control group was injected with normal saline following the same schedule. To confirm the successful establishment of PD models, motor coordination was assessed using the rotarod test, and mice that failed to meet the required performance criteria were excluded from further studies. The remaining mice were then randomly divided into four groups: control group (healthy mice), PD group (MPTP‐induced PD mice), DFP‐treated group (PD mice receiving 1 mM DFP via intranasal administration), and GQNPs‐treated group (PD mice receiving 1.5 mg/mL GQNPs via intranasal administration). For treatment, intranasal administration was performed every other day for three sessions following MPTP injections. The PD and control groups received normal saline instead of therapeutic treatment. Specifically, no optimal dose has been reported for intranasal delivery of gallium‐based nanoparticles. Therefore, the dose used in this study was selected mainly based on reported data from intravenous administration of gallium‐based nanoparticles [33]. Although this approach does not fully account for differences in intranasal delivery efficiency, the dosage used in this study was intended for a preliminary evaluation of the feasibility of this strategy. The same limitation also applies to intranasal DFP administration. However, in this study, DFP mainly served as a positive control and did not affect the evaluation of the anti‐ferroptotic effects of GQNPs. Behavioral tests were conducted 3 days after the final treatment to evaluate motor and cognitive functions. Finally, all mice were euthanized for further pathological and biochemical analyses.

### 5.16 Rotarod Test

Each mouse was placed on a rotating rod apparatus with a gradually increasing speed from 15 to 25 rpm and then to 30 rpm, with a maximum test duration of 300 s. The specific parameters were as follows: an initial speed of 15 rpm with an acceleration time of 20 s and a duration of 40 s; a second speed of 25 rpm with an acceleration time of 20 s and a duration of 40 s; and a third speed of 30 rpm with an acceleration time of 20 s and a duration of 160 s. The time each mouse remained on the rotating rod before falling was recorded. After an initial training period to ensure acclimatization, each mouse was tested at least three times.

### 5.17 Open Field Test

Before the open field test, each mouse was allowed to acclimate to the testing environment for 30 min. The open field apparatus consisted of a 50 cm × 50 cm × 40 cm enclosed arena. Each mouse was individually placed in the central region of the arena and monitored for 5 min using the Visutrack animal behavior analysis system. The following behavioral parameters were recorded and analyzed: total distance traveled, mean speed, total resting time, and the number of central zone transitions.

### 5.18 Morris Water Maze Test

Each mouse was allowed to acclimate to the experimental environment for 30 min before the Morris water maze test. The water maze consisted of a circular pool with a diameter of 120 cm, which was divided into four quadrants. A hidden platform was placed in one quadrant and submerged approximately 1 cm below the water surface, with the water temperature maintained at approximately 20°C throughout the experiment. The test was divided into two phases: a training phase on Days 1–4 and a test phase on Day 5. During the training phase, mice were placed into the pool from different quadrants each day and were given 1 min to locate the hidden platform. A successful escape was recorded when a mouse reached the platform and remained there for at least 3 s. After a successful escape, the mouse was allowed to stay on the platform for 10 s before being removed. If a mouse failed to find the platform within 1 min, the experimenter guided it to the platform, where it remained for 10 s to reinforce spatial learning. On Day 5, during the test phase, the platform was removed, and mice were placed into the pool from the quadrant opposite the previous platform location. Their swimming paths were recorded for 1 min, and the latency to reach the former platform location was used as an indicator of spatial memory and cognitive function.

### 5.19 Determination of TH, DA and MDA Concentrations

After the behavioral tests, mice were euthanized, and their midbrain tissues were carefully dissected on ice and weighed. Precooled 1× PBS was added at a volume corresponding to nine times the tissue weight, and the samples were homogenized using a mechanical homogenizer. The homogenates were then centrifuged at 4°C, and the supernatants were collected for further biochemical analysis. The concentrations of TH, DA, and MDA were determined using ELISA according to the manufacturer's instructions.

### 5.20 Immunofluorescence Staining and Biosafety Assessment

For biosafety assessment, blood samples were collected to analyze routine blood parameters, including WBCs, RBCs, HGB, PLT, as well as blood biochemical indices, including ALT, ALB, CREA, and TP levels. Additionally, major organs, including the brain, heart, liver, spleen, lung, and kidney, were harvested from mice in all treatment groups. Brain sections were subjected to TH immunofluorescence staining, whereas H&E staining was performed on all major organs to assess potential histopathological changes. Immunofluorescence and histological staining procedures were performed by AiFang Biological Co., Ltd.

### 5.21 Statistical Analysis

All data were analyzed using GraphPad Prism 10. Data are presented as the mean ± SD. Group sizes (n) for each experiment are provided in the corresponding figure legends. Experimenters were blinded to the treatment groups during both data collection and analysis. The allocation concealment was maintained until the statistical analysis was completed. Differences among multiple groups were analyzed using one‐way ANOVA followed by Tukey's post hoc test for multiple comparisons.

To ensure the validity of the behavioral assessments, the assumptions of normality and homogeneity of variance were examined before statistical analysis. The Shapiro–Wilk test was used to assess the data normality, and the Brown–Forsythe test was used to assess homogeneity of variance. When the assumptions for parametric tests were met, comparisons among multiple groups were performed using one‐way ANOVA. If the normality assumption was not met, the Kruskal–Wallis test was used. Exact p values are reported in the figures. ^*^ *p* < 0.05, ^**^ *p* < 0.01, ^***^ *p* < 0.001, ^****^ *p* < 0.0001, and ns indicates not significant.

### 5.22 Animal Ethics Statement

All animal experiments were conducted in accordance with the institutional guidelines for the care and use of laboratory animals and were approved by the Ethics Committee of Soochow University (License No. 202407A0632).

## Author Contributions

**Keyang Xu**: investigation, writing – original draft, methodology, writing – review and editing. **Dandan Kou**: methodology, investigation. **Xiao Xiao**: investigation, validation. **Bingbing Bai**: validation, writing – review and editing. **Caide Liu**: writing – review and editing, validation. **Mengyao Wu**: methodology, software. **Yadan Shi**: methodology. **Mengdan Xu**: supervision. **Xiaoyi Cao**: methodology. **Haodi Qi**: methodology. **Shuhua Wu**: writing – review and editing, validation. **Jianfeng Zeng**: conceptualization, writing – review and editing, funding acquisition, project administration. **Mingyuan Gao**: conceptualization, funding acquisition, writing – review and editing, project administration.

## Funding

This work was supported by the National Natural Science Foundation of China (82222033, 82572396, 82130059, and 82172003), the Natural Science Foundation of Jiangsu Province (BK20255001), and Suzhou Fundamental Research Project (SJC2023001).

## Conflicts of Interest

The authors declare no competing interests.

## Supporting information




**Supporting File**: advs77025‐sup‐0001‐SuppMat.docx.

## Data Availability

The data that support the findings of this study are available from the corresponding author upon reasonable request.
