# Supplementary material for: Intranasal Delivery of Gallium–Quercetin Nanoparticles for Multi‐Target Ferroptosis Inhibition in Parkinson's Disease
Source: Adv Sci (Weinh). 2026 Aug 7;13(60):e77025. doi: 10.1002/advs.77025 (PMC13449542; doi:10.1002/advs.77025)
Supplement: Supplementary file 1 [file ADVS-13-e77025-s001.docx]

**Supporting Information**

Supporting Information is available from the Wiley Online Library or from the author.

Supporting Information

Intranasal Delivery of Gallium–Quercetin Nanoparticles for Multi-Target Ferroptosis Inhibition in Parkinson’s Disease

Keyang Xu, Dandan Kou, Xiao Xiao, Bingbing Bai, Caide Liu, Mengyao Wu, Yadan Shi, Mengdan Xu, Xiaoyi Cao, Haodi Qi, Shuhua Wu, Jianfeng Zeng*, and Mingyuan Gao*

This section includes: Figure S1 to S15

**Figure S1.** Schematic workflow of the experimental design of this study.

**Figure S2.** (a) Hydrodynamic size distribution of GQNPs over time in PBS and 10% FBS, (b) zeta potential of GQNPs, and (c) antioxidant capacity of GQNPs at different concentrations measured using the T-AOC assay, with Trolox used as a reference.

**Figure S3.** (a) Cell viability after 24 h of treatment with increasing concentrations of GQNPs, (b) cell viability after 24 h of treatment with different concentrations of MPP^+^.

**Figure S4.** (a) Cellular Ga content after incubation with GQNPs for different durations, **p < 0.01, (b) CLSM images showing the colocalization of GQNPs (red) with lysosomes (green) in SH-SY5Y cells after 4 h of incubation, nuclei were stained with Hoechst 33342 (blue), (c) fluorescence intensity profile along the white dashed line in panel (b), (d) Pearson’s correlation coefficient analysis based on the CLSM images. Scale bar: 20 μm.

**Figure S5.** (a) Relative GPX4 expression levels normalized to β-actin in different treatment groups, (b) relative NRF2 expression levels normalized to β-actin in different treatment groups. Data are presented as the mean ± SD (n = 3). **p < 0.01, ****p < 0.0001.

**Figure S6.** Intracellular iron content in PD cells treated with 20 μg/mL free Ga^3+^. ***p < 0.001.

**Figure S7.** Anti-ferroptotic effects of individual components, their physical mixture and GQNPs. (a) Representative CLSM images of intracellular ROS levels detected using DCFH-DA, (b) quantification of the mean fluorescence intensity of the CLSM images, (c) GSH level, (d) MDA level and (e) total intracellular iron content in different treatment groups. *p < 0.05, **p < 0.01, ***p < 0.001, ****p < 0.0001.

**Figure S8.** Mitochondrial ferrous iron in different treatment groups. Data are presented as the mean ± SD (n = 3). **p < 0.01.

**Figure S9.** Mitochondrial respiratory chain complex III activity in different treatment groups. Data are presented as the mean ± SD (n = 4). ****p < 0.0001.

**Figure S10.** IVIS fluorescence signals of GQNPs. Fluorescence intensity increased with GQNPs concentration, with water used as the negative control.

**Figure S11.** (a) Representative photograph of the rotarod test, (b) screening of successfully modeled PD mice based on rotarod performance, (c) stratification of PD mice into subgroups according to rotarod test results.

**Figure S12.** Trajectory maps and heatmaps of mice with different treatments in the open field test (n = 6).

**Figure S13.** Trajectory maps of mice with various treatments in Morris water maze test. (n = 5 in the DFP-treated group, n = 6 in the other groups).

**Figure S14.** (a) Number of crossings over the target platform location, (b) total swimming distance, (c) swimming speed and (d) immobility time in the Morris water maze test (n = 5 in the DFP-treated group, n = 6 in the other groups).

**Figure S15.** H&E staining images of major organs, including the heart, liver, spleen, lung, and kidney, collected from different groups of mice. Scale bar: 200 μm.
